# Ultrasound Biomicroscopy Observation of Suspicious Primary Angle Closure Combined with the Relaxation of Zonule

**DOI:** 10.1155/2022/1614678

**Published:** 2022-03-08

**Authors:** Jingjing Ma, Nan Jiang, Zhongtai Jiang, Jing Lin, Cui Li, Guiqiu Zhao

**Affiliations:** Department of Ophthalmology, The Affiliated Hospital of Qingdao University, Qingdao 266003, Shandong, China

## Abstract

**Purpose:**

To investigate the difference in anterior segment parameters between suspicious primary angle closure (PACS) patients and normal patients as assessed by ultrasound biomicroscopy (UBM).

**Methods:**

From June 2019 to November 2020, 39 patients (50 eyes) with PACS in the Ophthalmology Department of Qingdao University Affiliated Hospital who underwent phacoemulsification and intraocular lens implantation were selected as the PACS group. 32 patients (50 eyes) who underwent phacoemulsification and intraocular lens implantation were selected as the normal group. In addition to routine preoperative examinations such as visual acuity, noncontact intraocular pressure, axis length (AL), and ocular B-ultrasound examination, UBM examinations were also performed, including measuring the central anterior chamber depth (ACD), the maximum transverse diameter of the ciliary process at both ends (STS), the vertical distance between the anterior apex of the lens and the maximum transverse diameter at both ends of the ciliary processes (h), and angle opening distance (AOD500), iris-zonule distance (IZD), trabecular-ciliary process distance (TCPD), trabecular-iris angle (TIA), iris thickness (IT), trabecular-ciliary process angle (TCPA), and anterior placement of the ciliary body (APCB) at four quadrants (superior, nasal, inferior, and temporal quadrants).

**Results:**

Compared with the normal group, the PACS group showed statistically differences in AL, ACD, h, ACD/AL, h/STS, IZD, AOD500, TCPD, TIA, TCPA, and APCB (*P* < 0.05), and there were no significant differences in STS and IT between the two groups (*P* > 0.05). In the PACS group, there were significant differences in AL, ACD, h, ACD/AL, h/STS, IZD, TCPD, TCPA, and APCB between PACS patients with zonular relaxation and without zonular relaxation (*P* < 0.05), while there were no significant differences in STS, AOD500, TIA, and IT (*P* > 0.05).

**Conclusion:**

UBM quantitatively enables to identify the anterior segment morphology, especially the zonules in patients of suspicious primary angle closure combined with the relaxation of zonule. Accurate measurement of UBM can be used to predict whether patients with PACS are combined with zonular relaxation, so as to provide a clinical imaging evidence for the diagnosis.

## 1. Introduction

Primary angle closure glaucoma (PACG) is one of the leading causes of blindness in Asians. The pupillary block, the plateau iris configuration, the lens induced mechanism, and ciliary ring block mechanism are the causes resulting in the closed angle. Relative pupillary block is considered the primary mechanism for angle closure [1–3]. In these cases, nonpupillary block mechanisms, such as lens induced, plateau iris, and peripheral angle crowding, may be involved [4–7]. Zonule is the elastic tissue that connects the lens and the ciliary body, which plays an important role in ensuring the centrality of the lens. Previous research studies have found that with increasing age, the number of zonule fibers become less, the attachment position becomes smaller, and the tension decreases. Subsequently, the zonule also becomes relax, and the position of fixed lens is unstable. Causes such as postural change result in the lens to move forward. Thus, pupillary block and angle closure occur, resulting in a shallower anterior chamber and increased intraocular pressure [8].

In recent years, many patients have been found to have zonular relaxation in the process of phacoemulsification, which increases the risk of surgery. In clinics, some patients have latent zonular relaxation, and some routine preoperative examinations cannot determine the abnormality of the lens zonule, so that the diagnosis and treatment plan cannot be accurately formulated [6, 7, 9]. Therefore, we have carried out the detailed measurement and analysis of some UBM parameters of PACS patients, in order to estimate whether the patients have zonular relaxation [10].

## 2. Methods

We retrospectively evaluated 39 PACS patients (50 eyes) and 32 normal patients (50 eyes) who underwent phacoemulsification combined with intraocular lens implantation at the Ophthalmology Department of the Affiliated Hospital of Qingdao University, between June 2019 and November 2020. The study was conducted in accordance with the Declaration of Helsinki. At all preoperative visits, all patients had standard UBM examinations, and abnormalities of the lens zonules were observed and recorded. The PACS was defined as an eye having appositional contact between iris and posterior trabecular meshwork for at least 180° on gonioscopy, with absence of peripheral anterior synechiae, or glaucomatous optic neuropathy, or visual field changes compatible with glaucoma; intraocular pressure did not exceed 21 mmHg [11, 12]. Subjects with secondary angle closure glaucoma, the history of intraocular surgery or penetrating eye injury, active keratitis or cornel keratopathy, intumescent cataract, evidence of a prior acute angle closure attack, and vitreoretinal diseases were excluded.

### 2.1. UBM Examination

The examination instrument was the MD-300L ophthalmic ultrasound biomicroscope. All patients were placed in the supine position and topical anesthesia with oxybuprocaine hydrochloride eye drops. The procedure was performed by an experienced examiner, who was positioned on the patient's right side. After the patient's head was gently steadied by an assistant, the appropriate eye cup was selected and placed gently in the conjunctival sac, poured proper amount of sterilized water into the eye cup, and placed the probe close to the eye in the cup. The patients were instructed to rotate the eyes at four quadrants (superior, nasal, inferior, and temporal quadrants) [8, 13]. The examiner observed the machine screen while scanning, adjusted the scanning direction and position to obtain the best image, and images of the four quadrants were obtained. Then, scanning images of four quadrants (superior, nasal, inferior, and temporal quadrants) were used for analysis [14]. Images obtained were graded quantitatively in all the 4 quadrants by an examiner with a special caliper in the UBM software. The scleral spur was identified, based on the differential tissue density between the collagen fibers of the scleral spur and the longitudinal muscle of the ciliary body. For the sake of uniformity, temporal quadrants UBM measurements of all angles were included in the analysis. The UBM parameters measured in the current study have been defined by Pavlin et al. [15]. All the measurements of linear parameters were expressed in millimeters and angular parameters in degrees.

- Central anterior chamber depth (ACD) was measured from the corneal endothelium to the anterior lenticular surface centered over the pupil
- h was the vertical distance from the vertex of the anterior surface of the lens to the largest transverse diameter of the ciliary process at both ends
- STS was the maximum transverse diameter between the ciliary processes at both ends
- Angle opening distance 500 (AOD500) was the distance between the inner corneal surface and the anterior iris surface measured on a line perpendicular to the plane of the trabecular meshwork at 500 *μ*m from the scleral spur
- Trabecular-ciliary process distance (TCPD) was measured on a line extending from the corneal endothelium at 500 *μ*m from the scleral spur passing perpendicularly through iris to the ciliary process
- Iris-zonule distance (IZD) was measured along the line of TCPD, from the posterior iris surface to the first visible zonule fiber at the point just clearing the ciliary process
- Trabecular-iris angle (TIA) was measured with the apex of the angle at the iris recess and the arms of the angle passing through a point on the trabecular meshwork at 500 *μ*m from the scleral spur and the point on the iris perpendicularly opposite
- Trabecular-ciliary process angle (TCPA) was measured with its apex at the scleral spur, one arm along the posterior corneal surface, and another arm along the most anterior surface of the ciliary body
- Iris thickness (IT) was measured at 500 *μ*m from the iris root
- Anterior placement of the ciliary body (APCB) was the distance from the most anterior point of the ciliary body to the vertical line from the inner wall of the sclera through the scleral spur

### 2.2. Statistical Analysis

Statistical analyses were conducted using SPSS version 19.0. Parameters between the PACS group and the normal group and PACS patients with and without zonular relaxation were compared using the independent *t*-test according to the distribution of the data. *P* < 0.05 indicated that the difference was statistically significant.

## 3. Results

The basic information (age, gender, and eye category) of the PACS group and the normal group is given in Table 1. In the PACS group, there were 39 patients (50 eyes) aged from 32 to 78 years old, with an average age of 60.96 ± 10.02 years old, including 17 male (22 eyes) and 22 female (28 eyes). In the normal group, there were 32 patients (50 eyes) aged from 35 to 75 years old, with an average age of 57.24 ± 11.23 years old, including 19 male (31 eyes) and 13 female (19 eyes). There were no statistically significant differences in age, gender, and eye category between the PACS group and the normal group (*P* > 0.05). In the PACS group, PACS with zonular relaxation were 15 patients (16 eyes) aged from 32 to 73 years old, with an average age of 57.13 ± 11.43 years old, including 8 male (9 eyes) and 7 female (7 eyes). PACS patients without zonular relaxation were 24 patients (34 eyes) aged from 42 to 78 years old, with an average of 62.76 ± 8.90 years old, including 9 male (13 eyes) and 15 female (21 eyes). The results suggested that there were no significant differences in age, gender, and eye category between PACS with zonular relaxation and PACS without zonular relaxation (*P* > 0.05).

The results of clinical data are given in Table 2. We first compared the difference in ACD, AL, h, STS, ACD/AL, and h/STS between the normal group and the PACS group, as well as PACS patients with and without zonular relaxation. The ACD in the PACS group was significantly shorter than in the normal group (1.92 ± 0.26 VS 2.86 ± 0.39 mm, *P* < 0.05), and ACD/AL in the PACS group was significantly smaller than that in the normal group (0.08 ± 0.01 vs. 0.12 ± 0.02, *P* < 0.05). The AL (24.66 ± 1.02 vs. 23.93 ± 0.82 mm, *P* < 0.05), h (1.95 ± 0.27 vs. 1.50 ± 0.26 mm, *P* < 0.05), and h/STS (0.18 ± 0.02 vs. 0.14 ± 0.02, *P* < 0.05) were significantly larger in the PACS group than in the normal group. However, there was no significant difference in STS (11.06 ± 0.64 vs. 10.87 ± 0.78 mm, *P* > 0.05) between the two groups. In PACS with zonular relaxation, ACD was significantly shorter than in PACS without zonular relaxation (1.78 ± 0.31 vs. 1.99 ± 0.20 mm, *P* < 0.05). The ACD/AL in PACS with zonular relaxation was significantly smaller than in PACS without zonular relaxation (0.07 ± 0.01 vs. 0.08 ± 0.01, *P* < 0.05). The AL (25.64 ± 0.62 vs. 24.20 ± 0.83 mm, *P* < 0.05), h (2.11 ± 0.24 vs. 1.88 ± 0.25 mm, *P* < 0.05), and h/STS (0.19 ± 0.02 vs. 0.17 ± 0.03, *P* < 0.05) were significantly larger in PACS with zonular relaxation than in PACS without relaxation. In the PACS group, there was no statistically significant difference in STS between PACS with zonular relaxation and PACS without zonular relaxation (11.28 ± 0.71 vs. 10.96 ± 0.58 mm, *P* > 0.05). Second, the results of some UBM parameters (IZD, AOD500, TCPD, TIA, TCPD, IT, and APCB) at four quadrants (superior, nasal, inferior, and temporal quadrants) are given in Table 2. The IZD (*P* < 0.05), AOD500 (*P* < 0.05), and TCPD (*P* < 0.05) at four quadrants (superior, nasal, inferior, and temporal quadrants) were significantly shorter in the PACS group than in the normal group. The TIA (*P* < 0.05) and TCPA (*P* < 0.05) at four quadrants (superior, nasal, inferior, and temporal quadrants) were significantly narrower in the PACS group than in the normal group. The APCB (*P* < 0.05) at four quadrants (superior, nasal, inferior, and temporal quadrants) were significantly larger in the PACS group than in the normal group. There were no significant differences in IT (*P* > 0.05) at four quadrants (superior, nasal, inferior, and temporal quadrants) between the two groups. In the PACS group, the PACS patients with zonular relaxation had significantly shorter IZD (*P* < 0.05) at four quadrants (superior, nasal, inferior, and temporal quadrants), significantly shorter TCPD (*P* < 0.05) at four quadrants (superior, nasal, inferior, and temporal quadrants), significantly narrower TCPA (*P* < 0.05) at four quadrants (superior, nasal, inferior, and temporal quadrants), and significantly larger APCB (*P* < 0.05) at four quadrants (superior, nasal, inferior, and temporal quadrants) than PACS without zonular relaxation. There were no significant differences in AOD500 (*P* > 0.05), TIA (*P* > 0.05), and IT (*P* > 0.05) at four quadrants (superior, nasal, inferior, and temporal quadrants) between PACS with zonular relaxation and PACS without zonular relaxation.

## 4. Discussion

This study compared the difference of zonule between the PACS group and the normal group by measuring some parameters on UBM images. The zonule is located behind the iris, around the equator of the lens, and inside the ciliary body. It is the tissue connecting the equatorial and ciliary body of the lens. It has been reported that the role of the zonule is to fix the position of the lens. After the stability of the lens changes due to the abnormality of the zonule, the smooth progress of cataract surgery can be affected [16, 17]. In serious cases, the angle of the chamber will become narrower and the intraocular pressure will become higher, which will adversely affect the patient [18, 19]. Therefore, it is very important to evaluate the condition of the patient eyes' zonule before operation. UBM is a new type of examination instrument with high resolution, and it has been widely used in preoperative examination of cataract surgery. Studies have found that UBM can accurately display the patients' zonule, which is helpful for doctors to understand the patients' eyes condition and make appropriate surgical plan, so as to reduce the risk of surgery [20–22]. In addition, UBM is a contact inspection. Due to the gravity of the liquid in the eye cup and the pressure of the eye cup itself on the surface of the eyeball, it is easy to cause the eyeball to deform, and the measurement parameters may cause errors.

This study included the PACS patients and the normal people who had normal intraocular pressure before surgery and planned to undergo phacoemulsification combined with intraocular lens implantation. We quantitatively collected the basic information and some UBM parameters of the PACS group and the normal group [8, 23–26]. The results showed that compared with the normal group, the parameters other than STS and IT were different in the PACS group. Among them, ACD and ACD/AL were significantly smaller in the PACS group than in the normal group, AL, h, and h/STS were significantly larger in the PACS group than in the normal group, IZD, AOD500, and TCPD were significantly shorter in the PACS group than in the normal group at four quadrants (superior, nasal, inferior, and temporal quadrants), TIA and TCPA were significantly narrower in the PACS group than in the normal group at four quadrants (superior, nasal, inferior, and temporal quadrants), and APCB was significantly larger in the PACS group than in the normal group at four quadrants (superior, nasal, inferior, and temporal quadrants). In the PACS group, ACD and ACD/AL were significantly smaller in PACS with zonular relaxation than in PACS without zonular relaxation. Besides, AL, h, and h/STS were significantly larger in PACS with zonular relaxation than in PACS without zonular relaxation, IZD and TCPD were significantly shorter in PACS with zonular relaxation than in PACS without zonular relaxation at four quadrants (superior, nasal, inferior, and temporal quadrants), TCPA was narrower in PACS with zonular relaxation than in PACS without zonular relaxation at four quadrants (superior, nasal, inferior, and temporal quadrants), and APCB was narrower in PACS with zonular relaxation than in PACS without zonular relaxation at four quadrants (superior, nasal, inferior, and temporal quadrants). However, there was no statistically significant difference in STS, IT, AOD500, and TIA at four quadrants (superior, nasal, inferior, and temporal quadrants) between the two groups.

It was suggested that preoperative UBM examination was performed on the operative eye of the patient and could reflect the condition of the patient's zonule, so as to make a better surgical plan.

To sum up, we can measure ACD, h, STS, IZD, AOD500, TCPD, TIA, TCPA, and APCB of the operative eye by UBM to predict whether the patient has zonule relaxation, so as to choose the best treatment. However, there are some limitations in this study, that is, the sample size is relatively small, so it is still necessary to expand the sample size for further study.

## Figures and Tables

**Table 1:** Basic information of study subjects.

	PACS *n* = 50		Normal *n* = 50	*P* value^a^	
	PACS with zonular relaxation *n* = 16	PACS without zonular relaxation *n* = 34		PACS with zonular relaxation vs. PACS without zonular relaxation	PACS vs. normal
Mean age ± SD, y	60.96 ± 10.02				
	57.13 ± 11.43	62.76 ± 8.90	57.24 ± 11.23	*P* = 0.095	*P* = 0.084
Sex, men/women	17/22				
	8/7	9/15	19/13	*P* = 0.332	*P* = 0.186
Eye (right/left)	25/25				
	9/7	16/18	26/24	*P* = 0.544	*P* = 0.084

PACS, primary angle closure suspect. ^a^*P* value for comparison between PACS with normal and PACS with zonular relaxation with PACS without zonular relaxation.

**Table 2:** Comparison of parameters among 16 PACS with zonular relaxation, 34 PACS without zonular relaxation, and 50 normal.

		PACS *n* = 50		Normal *n* = 50	*P* value^a^	
		PACS with zonular relaxation, *n* = 16	PACS without zonular relaxation, *n* = 34		PACS with zonular relaxation vs. PACS without zonular relaxation	PACS vs. normal
ACD		1.92 ± 0.26				
		1.78 ± 0.31	1.99 ± 0.20	2.86 ± 0.39	*P* < 0.05	*P* < 0.05
AL		24.66 ± 1.02				
		25.64 ± 0.62	24.20 ± 0.83	23.93 ± 0.82	*P* < 0.05	*P* < 0.05
h		1.95 ± 0.27				
		2.11 ± 0.24	1.88 ± 0.25	1.50 ± 0.26	*P* < 0.05	*P* < 0.05
STS		11.06 ± 0.64				
		11.28 ± 0.71	10.96 ± 0.58	10.87 ± 0.78	*P* > 0.05	*P* > 0.05
ACD/AL		0.08 ± 0.01				
		0.069 ± 0.01	0.08 ± 0.01	0.119 ± 0.02	*P* < 0.05	*P* < 0.05
h/STS		0.18 ± 0.02				
		0.187 ± 0.02	0.17 ± 0.03	0.138 ± 0.02	*P* < 0.05	*P* < 0.05
IZD	Superior	0.41 ± 0.10				
		0.37 ± 0.06	0.43 ± 0.11	0.55 ± 0.09	*P* < 0.05	*P* < 0.05
	Nasal	0.38 ± 0.10				
		0.34 ± 0.08	0.40 ± 0.11	0.50 ± 0.09	*P* < 0.05	*P* < 0.05
	Inferior	0.40 ± 0.08				
		0.36 ± 0.07	0.41 ± 0.08	0.50 ± 0.08	*P* < 0.05	*P* < 0.05
	Temporal	0.36 ± 0.09				
		0.33 ± 0.06	0.38 ± 0.08	0.49 ± 0.08	*P* < 0.05	*P* < 0.05
AOD500	Superior	0.15 ± 0.07				
		0.15 ± 0.03	0.16 ± 0.08	0.29 ± 0.08	*P* > 0.05	*P* < 0.05
	Nasal	0.20 ± 0.10				
		0.22 ± 0.07	0.19 ± 0.11	0.33 ± 0.10	*P* > 0.05	*P* < 0.05
	Inferior	0.18 ± 0.07				
		0.20 ± 0.05	0.17 ± 0.08	0.31 ± 0.10	*P* > 0.05	*P* < 0.05
	Temporal	0.21 ± 0.18				
		0.22 ± 0.07	0.18 ± 0.09	0.36 ± 0.10	*P* > 0.05	*P* < 0.05
TCPD	Superior	1.11 ± 0.12				
		1.07 ± 0.07	1.12 ± 0.123	1.40 ± 0.15	*P* < 0.05	*P* < 0.05
	Nasal	1.11 ± 0.13				
		1.06 ± 0.08	1.14 ± 0.136	1.36 ± 0.15	*P* < 0.05	*P* < 0.05
	Inferior	1.13 ± 0.12				
		1.09 ± 0.08	1.15 ± 0.131	1.39 ± 0.14	*P* < 0.05	*P* < 0.05
	Temporal	1.09 ± 0.09				
		1.05 ± 0.72	1.11 ± 0.10	1.37 ± 0.14	*P* < 0.05	*P* < 0.05
TIA	Superior	18.06 ± 6.21				
		19.52 ± 5.39	17.37 ± 6.52	29.24 ± 7.53	*P* > 0.05	*P* < 0.05
	Nasal	21.01 ± 9.65				
		23.83 ± 8.33	19.68 ± 10.05	33.29 ± 9.93	*P* > 0.05	*P* < 0.05
	Inferior	18.89 ± 6.84				
		20.03 ± 5.75	18.35 ± 7.31	31.91 ± 7.99	*P* > 0.05	*P* < 0.05
	Temporal	20.67 ± 9.30				
		23.89 ± 7.18	19.15 ± 9.88	35.01 ± 10.39	*P* > 0.05	*P* < 0.05
TCPA	Superior	67.64 ± 12.55				
		62.66 ± 10.04	69.99 ± 13.05	86.61 ± 12.64	*P* < 0.05	*P* < 0.05
	Nasal	67.53 ± 12.33				
		63.11 ± 7.91	69.61 ± 13.55	87.12 ± 13.73	*P* < 0.05	*P* < 0.05
	Inferior	69.63 ± 12.40				
		64.69 ± 7.95	71.96 ± 13.49	87.34 ± 14.17	*P* < 0.05	*P* < 0.05
	Temporal	68.06 ± 10.74				
		62.44 ± 8.66	70.70 ± 10.71	87.41 ± 13.33	*P* < 0.05	*P* < 0.05
IT	Superior	0.39 ± 0.07				
		0.38 ± 0.05	0.40 ± 0.08	0.41 ± 0.07	*P* > 0.05	*P* > 0.05
	Nasal	0.41 ± 0.08				
		0.41 ± 0.08	0.41 ± 0.07	0.43 ± 0.07	*P* > 0.05	*P* > 0.05
	Inferior	0.43 ± 0.07				
		0.42 ± 0.08	0.44 ± 0.07	0.45 ± 0.09	*P* > 0.05	*P* > 0.05
	Temporal	0.42 ± 0.07				
		0.42 ± 0.07	0.41 ± 0.07	0.44 ± 0.07	*P* > 0.05	*P* > 0.05
APCB	Superior	0.53 ± 0.08				
		0.62 ± 0.03	0.48 ± 0.04	0.33 ± 0.04	*P* < 0.05	*P* < 0.05
	Nasal	0.54 ± 0.07				
		0.63 ± 0.04	0.50 ± 0.03	0.37 ± 0.05	*P* < 0.05	*P* < 0.05
	Inferior	0.54 ± 0.07				
		0.63 ± 0.03	0.50 ± 0.03	0.38 ± 0.04	*P* < 0.05	*P* < 0.05
	Temporal	0.55 ± 0.07				
		0.63 ± 0.03	0.51 ± 0.04	0.38 ± 0.04	*P* < 0.05	*P* < 0.05

ACD, central anterior chamber depth; AL, axial length; IZD, iris-zonule distance; AOD500, angle opening distance 500; TCPD, trabecular-ciliary process distance; TIA, trabecular-iris angle; TCPA, trabecular-ciliary process angle; IT, iris thickness; APCB, anterior placement of the ciliary body. Values, except for *P* values, are expressed as mean ± standard deviation.

## Data Availability

The data used to support the findings of this study are available from the corresponding author upon request and are not publicly available due to privacy or ethical restrictions.

## References

[1] Seland J. H. (1992). The lens capsule and zonulae. *Acta ophthalmologica. Supplement*.

[2] Fontana S. T., Brubaker R. F. (1980). Volume and depth of the anterior chamber in the normal aging human eye. *Archives of Ophthalmology*.

[3] Farnsworth P. N., Shyne S. E. (1979). Anterior zonular shifts with age. *Experimental Eye Research*.

[4] Assia E. I., Apple D. J., Morgan R. C., Legler U. F., Brown S. J. (1991). The relationship between the stretching capability of the anterior capsule and zonules. *Investigative Ophthalmology & Visual Science*.

[5] Gomes Prado V., Dorairaj S., Gustavo Biteli L. (2014). Role of laser iridoplasty in the management of angle closure mechanisms other than pupillary block. *Journal of Current Glaucoma Practice*.

[6] Zeng Y., Liu Y., Liu X. (2009). Comparison of lens thickness measurements using the anterior segment optical coherence tomography and A-scan ultrasonography. *Investigative Opthalmology & Visual Science*.

[7] Taketani F., Matuura T., Yukawa E., Hara Y. (2004). Influence of intraocular lens tilt and decentration on wavefront aberrations. *Journal of Cataract & Refractive Surgery*.

[8] Mansoori T., Balakrishna N. (2017). Anterior segment morphology in primary angle closure glaucoma using ultrasound biomicroscopy. *Journal of Current Glaucoma Practice*.

[9] Shen M., Cui L., Li M., Zhu D., Wang M. R., Wang J. (2011). Extended scan depth optical coherence tomography for evaluating ocular surface shape. *Journal of Biomedical Optics*.

[10] Hirasawa H., Tomidokoro A., Kunimatsu S. (2009). Ultrasound biomicroscopy in narrow peripheral anterior chamber eyes with or without peripheral anterior synechiae. *Journal of Glaucoma*.

[11] Holladay J. T., Piers P. A., Koranyi G., van der Mooren M., Norrby N. E. S. (2002). A new intraocular lens design to reduce spherical aberration of pseudophakic eyes. *Journal of Refractive Surgery*.

[12] Razeghinejad M. R., Myers J. S. (2018). Contemporary approach to the diagnosis and management of primary angle-closure disease. *Survey of Ophthalmology*.

[13] Henzan I. M., Tomidokoro A., Uejo C. (2011). Comparison of ultrasound biomicroscopic configurations among primary angle closure, its suspects, and nonoccludable angles: the Kumejima Study. *American Journal of Ophthalmology*.

[14] Bezzina A. M. (1992). Ultrasound biomicroscopy of anterior segment structures in normal and glaucomatous eyes. *American Journal of Ophthalmology*.

[15] Pavlin C. J., Harasiewicz K., Foster F. S. (1992). Ultrasound biomicroscopy of anterior segment structures in normal and glaucomatous eyes. *American Journal of Ophthalmology*.

[16] Ikeda N., Ikeda T., Nagata M., Mimura O. (2002). Pathogenesis of transient high myopia after blunt eye trauma. *Ophthalmology*.

[17] Khurana M., Shah D. D., George R. J., Vijaya L., Balekudaru S. (2020). Phacoemulsification in eyes with long anterior zonules. *Journal of Cataract & Refractive Surgery*.

[18] Thomas R., Parikh R., Muliyil J., Kumar R. S. (2003). Five-year risk of progression of primary angle closure to primary angle closure glaucoma: a population-based study. *Acta Ophthalmologica Scandinavica*.

[19] Xing X. J., Tang X., Song H., Li W. W. (2010). [Comparison of tilt and decentration of four different kinds of aspheric intraocular lenses implantat ion]. *Zhonghua Yan Ke Za Zhi*.

[20] Spaeth G. L., Azuara-Blanco A., Araujo S. V., Augsburger J. J. (1997). Intraobserver and interobserver agreement in evaluating the anterior chamber angle configuration by ultrasound biomicroscopy. *Journal of Glaucoma*.

[21] Ishikawa H., Liebmann J. M., Ritch R. (2000). Quantitative assessment of the anterior segment using ultrasound biomicroscopy. *Current Opinion in Ophthalmology*.

[22] Dorairaj S., Liebmann J. M., Ritch R. (2007). Quantitative evaluation of anterior segment parameters in the era of imaging. *Transactions of the American Ophthalmological Society*.

[23] Tello C., Liebmann J., Potash S. D., Cohen H., Ritch R. (1994). Measurement of ultrasound biomicroscopy images: intraobserver and interobserver reliability. *Investigative Ophthalmology & Visual Science*.

[24] Jiang Y., He M., Huang W., Huang Q., Zhang J., Foster P. J. (2010). Qualitative assessment of ultrasound biomicroscopic images using standard photographs: the liwan eye study. *Investigative Opthalmology & Visual Science*.

[25] Kwon J., Sung K. R., Han S., Moon Y. J., Shin J. W. (2017). Subclassification of primary angle closure using anterior segment optical coherence tomography and ultrasound biomicroscopic parameters. *Ophthalmology*.

[26] Subbiah S., Thomas P. A., Nelson Jesudasan C. A. (2017). Comparison of ultrasound biomicroscopy and ultrasonographic parameters in eyes with phacomorphic glaucoma and eyes with mature cataract. *International Ophthalmology*.

